# Prolonged silencing of diacylglycerol acyltransferase‐1 induces a dedifferentiated phenotype in human liver cells

**DOI:** 10.1111/jcmm.12685

**Published:** 2015-10-23

**Authors:** Soyoung Chang, Pil Soo Sung, Jungsul Lee, Junseong Park, Eui‐Cheol Shin, Chulhee Choi

**Affiliations:** ^1^ Department of Bio and Brain EngineeringKAISTDaejeonKorea; ^2^ Laboratory of Immunology and Infectious DiseasesGraduate School of Medical Science and EngineeringKAISTDaejeonKorea; ^3^ KAIST Institute for the BioCenturyKAISTDaejeonKorea

**Keywords:** diacylglycerol acyltransferase‐1, gene silencing, stem cell‐like phenotype, cirrhosis

## Abstract

Diacylglycerol acyltransferase‐1 (DGAT1), a key enzyme in triglyceride (TG) biogenesis, is highly associated with metabolic abnormalities, such as obesity and type 2 diabetes. However, the effects of DGAT1 silencing in the human liver have not been elucidated. To investigate the effects of DGAT1 silencing in human liver cells, we compared the cellular behaviours of DGAT1‐deficient Huh‐7.5 cell lines with those of control Huh‐7.5 cells. DGAT1‐deficient cells acquired dedifferentiated and stem cell‐like characteristics, such as formation of aggregates in the presence of high levels of growth factors, high proliferation rates and loss of albumin secretion. In relation to aggregate formation, the expression level of various adhesion molecules was significantly altered in DGAT1‐deficient cells. Microarray data analysis and immunostaining of patient tissue samples clearly showed decreased expression levels of DGAT1 and integrin β1 in patients who have nodular cirrhosis without fatty degeneration.

## Introduction

Diacylglycerol acyltransferase‐1 (DGAT1) is a key enzyme that plays an important role in the final step of TG biosynthesis 1, 2. Diacylglycerol acyltransferase‐1 was recently considered a therapeutic target for metabolic diseases, such as type 2 diabetes and obesity, as excessive accumulation of TG is frequently associated with these diseases 3.

The human liver is the major organ for TG synthesis and DGAT1 is abundantly expressed therein. However, the effects of DGAT1 inhibition or DGAT1 silencing in human hepatocytes are unknown, although DGAT1 inhibitors have been developed and are undergoing clinical trials for the treatment of metabolic diseases 4. This is because most DGAT1 studies have used the mice model and DGAT1 is only weakly expressed in the mouse liver 1, 5. Diacylglycerol acyltransferase‐1 is mainly expressed in the intestines, skeletal muscles and heart of mice; whereas human DGAT1 is primarily expressed in the small intestine and liver 5.

Recently, DGAT1 was highlighted because it plays an important role in trafficking hepatitis C virus (HCV) core protein to lipid droplets 6. In addition, our group recently demonstrated that complete and long‐term silencing of DGAT1 in Huh‐7.5 cells inhibited HCV entry by down‐regulation of claudin‐1 7. We have established DGAT1‐silenced cell lines by gene deletion using the transcription activator‐like effector nuclease (TALEN) and shRNA‐lentivirus transduction, and investigated impaired HCV entry to DGAT1‐silenced cell lines 7. In this study, we investigated the phenotypic alterations caused by complete and long‐term DGAT1 silencing in two liver‐derived cell lines, Huh‐7.5 and HepG2 cell lines. The data indicated that DGAT1‐silenced cell lines exhibited dedifferentiated and stem cell‐like phenotypes.

Liver cirrhosis is defined as the development of regenerative nodules and fibrous bands by chronic liver injury, and it is well known that proliferative activity of regenerative hepatocytes is increased in cirrhotic liver 8, 9. We assumed that the decreased DGAT1 expression in cirrhotic liver might have an association with increased proliferative activity in regenerative hepatocytes, and demonstrated DGAT1 down‐regulation in patients with liver cirrhosis without fatty degeneration.

## Materials and methods

### Cell culture

The detailed cell culture method was described previously 7. Huh‐7.5 cells (Apath, LLC, Brooklyn, NY, USA) and HepG2 cells (ATCC, Manassas, VA, USA) were maintained in DMEM supplemented with 10% fetal bovine serum, 4.5 g/l glucose, L‐glutamine and 1% penicillin/streptomycin (Invitrogen, Carlsbad, CA, USA). shRNA‐harbouring cells were cultured in complete medium supplemented with 1 μg puromycin (Sigma‐Aldrich, St. Louis, MO, USA), and shRNA‐resistant DGAT1‐transfected, DGAT1 knock‐down cells were maintained in complete medium supplemented with 1 μg puromycin and 1 mg/ml G418 (A.G. Scientific, San Diego, CA, USA).

### Generation of DGAT1 knock‐down and knockout (KO) cell lines

Diacylglycerol acyltransferase‐1 knock‐down Huh‐7.5 and HepG2 cell lines were generated as described previously 7. Briefly, three different lentiviral constructs expressing validated sequences of shRNA‐DGAT1 were purchased. VSV‐G plasmid, gag‐pol plasmid and shRNA plasmid were transfected into 293TN cells, and lentiviral particles were harvested 48 hrs after transfection. Huh‐7.5 and HepG2 cells were transduced with lentivirus, and expanded over 3–6 weeks in puromycin‐containing selection medium.

Diacylglycerol acyltransferase‐1 KO Huh‐7.5 cells were generated as described previously 7. Huh‐7.5 cells were cotransfected with total 8 μg DNA comprising 2 μg plasmid encoding one member of *DGAT1*‐targeting TALEN, 2 μg plasmid encoding another member of the TALEN and 4 μg the magnetic reporter. After the magnetic separation, 62 single‐cell‐derived colonies were analysed using T7E1 assay and sequencing. Finally, a *DGAT1*‐knockout cell line was chosen harbouring a shifted open reading frame or premature stop codon in the *DGAT1* gene.

### Aggregate formation assay

Cells of each stable cell line were counted, seeded in six‐well plates and incubated for 48 hrs with complete medium or stem cell medium consisting of DMEM F/12 (Invitrogen) supplemented with bFGF, EGF and B‐27 supplements (Invitrogen). Adherent cells and aggregate cells were examined using a phase‐contrast microscopy with 40× magnification.

### Measurement of cell proliferation and apoptosis

Cell proliferation and apoptosis were assessed using the Apoptosis, DNA Damage and Cell Proliferation Kit (BD Transduction Laboratories, San Jose, CA, USA) according to the manufacturer's protocol using a method described previously 7. Briefly, 1 mM BrdU was administered to the control and DGAT1‐silenced cell lines for 3 hrs. After fixing and permeabilization, cells were stained with PerCP‐Cy^™^5.5‐conjugated mouse anti‐BrdU (BD Transduction Laboratories) and PE‐conjugated mouse anti‐cleaved PARP (Asp214) antibodies. The LSR II instrument (BD Transduction Laboratories) was used to detect fluorescence.

### Flow cytometry

For surface staining of E‐cadherin and N‐cadherin, a PE‐antimouse E‐cadherin antibody (clone 36/E‐cadherin; BD Transduction Laboratories) and PE‐antimouse N‐cadherin antibody (clone 8C11; BD Transduction Laboratories), respectively, were used. After incubation with the antibodies for 30 min at 4°C, cells were resuspended in FACS buffer. Up to 50,000 events were detected using the LSR II instrument (BD Transduction Laboratories). The FlowJo software (TreeStar, Ashland, OR, USA) was used for the data analysis.

### Enzyme‐linked immunosorbent assay

The supernatants from each cell line were collected after culturing for 24 hrs and processed using the Albumin Human ELISA kit (ab108788; Abcam, Cambridge, MA, USA) according to the manufacturer's instructions.

### RNA extraction, cDNA synthesis and real‐time quantitative PCR

Total RNA isolation, cDNA synthesis and TaqMan real‐time quantitative PCR were performed as described previously 7. For each gene, validated primer and probe sequences from the Taqman Gene Expression Assay (Applied Biosystems, Foster City, CA, USA) were purchased and used. All real‐time qPCR reactions were performed in triplicate and the data are presented as means ± S.E.M.

### Immunoblot analysis

Cell lysates (20 μg total protein) were separated in 10 or 12% SDS‐PAGE gels, then transferred to nitrocellulose membranes and probed with antibodies against DGAT1, hepatocyte nuclear factor 4α (HNF4α), integrin β1, integrin β2, integrin α6, N‐cadherin, E‐cadherin and GAPDH. To detect bound antibodies, the blots were developed using enhanced chemiluminescence reagents (AbFrontier, Seoul, Korea).

### Oil‐red O staining

Oil‐Red O staining was performed as previously described 10 with some modifications. shRNA‐control and DGAT1‐silenced cells were grown in 24‐well plate and fixed with 2% paraformaldehyde for 20 min. After fixation, cells were stained with 0.1% Oil‐Red O working solution (Sigma‐Aldrich) for 2 hrs at room temperature. Cells were washed extensively to remove dye precipitates, and visualized under light microscopy with 100× magnification. To quantify intracellular TG level, 100% isopropanol was added to each sample; after shaking at room temperature for 30 min, eluted samples were read at 500 nm on a spectrophotometer.

### Microarray database analysis

A total of 12 microarray gene expression profile data sets from Gene Expression Omnibus (GEO) was used in this analysis (Table 1). Data sets were selected according to annotations for each sample. We integrated the data sets by normalizing expression levels using the Universal expression Code (UPC) with the default normal‐normal model.

**Table 1:** Accession numbers of GEO data sets

GSE‐ID	Normal	NAFLD induced‐ cirrhosis	Viral hepatitis induced‐ cirrhosis	HCC
GSE6222	2			10
GSE6764	10		13	
GSE14668	20			
GSE19665	10			20
GSE33006	3			3
GSE41804	20			21
GSE28619	7			
GSE38941	10			
GSE38597	2			
GSE14323	19		41	38
GSE17548			20	17
GSE49541		32		
Total samples (*n*)	103			109

GEO: Gene Expression Omnibus; NAFLD: non‐alcoholic fatty liver disease; HCC: hepatocellular carcinoma.

### Immunohistochemistry

Paraffin‐embedded 5‐μm sections from tissue microarray slides of multiple liver diseases (LV1201; US Biomax, Rockville, MD, USA) were deparaffinized with xylene and rehydrated with alcohol. The slides were stained with anti‐DGAT1 and anti‐integrin β1 antibodies [diluted 1:100 in 1% bovine serum albumin (BSA) and 0.3% Triton X‐100]. After washing with PBS, the slides were incubated with FITC‐conjugated goat anti‐rabbit IgG (Santa Cruz Biotechnology, Santa Cruz, CA, USA) as the secondary antibody. Nuclei were counterstained with Hoechst 33342 (Invitrogen) for 15 min. After washing with PBS, the slides were mounted in Faramount aqueous mounting medium (DakoCytomation, Carpinteria, CA, USA) and the fluorescence signal was visualized using confocal or two‐photon microscopy (Carl Zeiss, Gottingen, Germany).

### Statistical analysis

The data are presented as means ± S.E.M. Levels of significance for comparisons between two independent samples were determined using Student's *t*‐test. Groups were compared by one‐way anova with Tukey's post hoc test applied to significant main effects.

## Results

### Dedifferentiated and stem cell‐like phenotype in DGAT1‐deficient Huh‐7.5 cell lines

**Figure 1:**
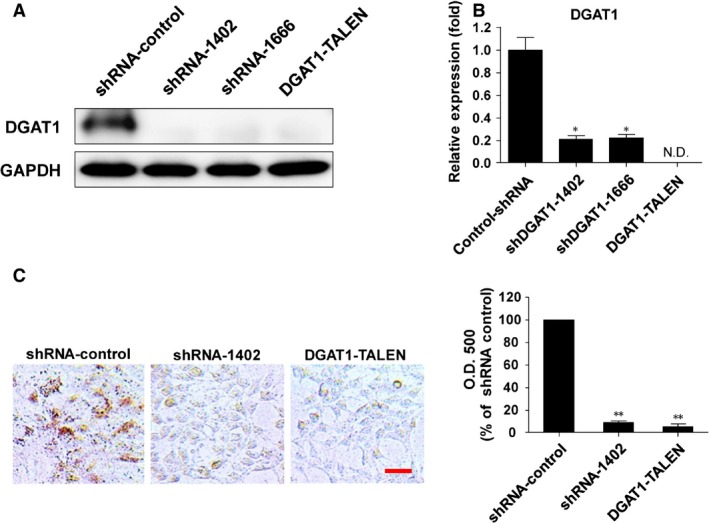
General characteristics of DGAT1‐deficient Huh‐7.5 cells. (**A**) DGAT1 protein level was examined by immunoblotting in DGAT1‐silenced cells and the control cell line. (**B**) DGAT1 mRNA level was determined by real‐time quantitative PCR in each cell line and normalized to β‐actin (n = 3). Data are presented as means ± S.E.M.; asterisks indicate significant differences by anova, **P < 0.01; N.D.: not detected. (**C**) Intracellular lipid droplets were quantitated using Oil‐red O staining in control and DGAT1‐silenced cells. OD values of eluted Oil‐red O with 100% isopropanol were measured and shown as with bar graphs; scale bar, 20 μm. Data are presented as means ± S.E.M.; asterisks indicate significant differences by anova, **P < 0.05.

As reported previously, DGAT1 expression was significantly knocked down by shRNA‐lentivirus transduction, or the *DGAT1* gene was knocked out by a pair of TALENs, in Huh‐7.5 cells 7. Diacylglycerol acyltransferase‐1 mRNA and protein levels were measured to confirm of DGAT1 silencing (Fig. 1A and B). We also confirmed that intracellular TG levels were decreased in DGAT1‐silenced cells (Fig. 1C).

**Figure 2:**
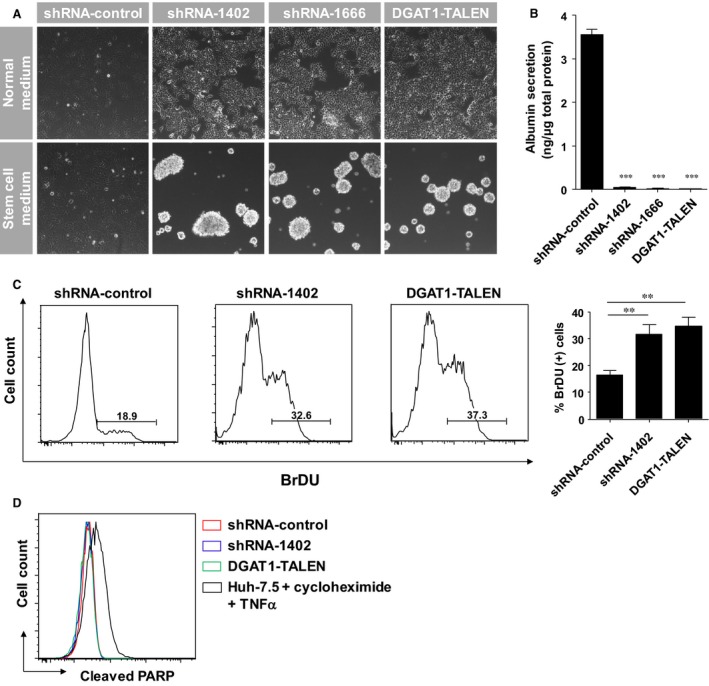
Phenotypic alterations caused by DGAT1 silencing in Huh‐7.5 cells. (**A**) DGAT1‐deficient cells and the control cell line were cultured in either complete medium or stem cell medium without serum. After 48 hrs incubation, cells were visualized using a phase‐contrast microscope. (**B**) The secreted albumin levels in the supernatants of each cell line after 24 hrs culture was determined by ELISA (n = 4). (**C**) Proliferation of each cell line was measured by BrdU incorporation assay. BrdU‐positive cells were counted by flow cytometry. Histograms are representative of three independent experiments and the proportions (%) of BrdU‐positive cells are shown as with bar graphs. (**D**) Histograms of cells expressing cleaved PARP are presented. A histogram of Huh‐7.5 cells treated with cycloheximide and TNFα is shown as a positive control. Data are presented as means ± S.E.M.; asterisks indicate significant differences by anova; **P < 0.01, ***P < 0.005.

During subsequent culture, the DGAT1‐silenced cell lines formed clusters similar to neurospheres; whereas the control parental cell line spread evenly in normal medium. To assess their ability to form spheroid, we cultivated DGAT1‐silenced cells in conventional stem cell culture medium containing high levels of growth factors without serum. After 24 hrs of incubation, DGAT1‐silenced cells were detached from the substrate and formed aggregates in stem cell culture medium; in contrast, the control cells grew as an adherent form (Fig. 2A). The production of albumin by DGAT1‐silenced cells was reduced significantly (Fig. 2B). These DGAT1‐silenced cells also showed higher proliferation than the control cells (Fig. 2C); however, the apoptosis rate of DGAT1‐silenced cells, as determined using cleaved PARP levels, was identical to that of the control parental cell line (Fig. 2D). These results indicate that DGAT1‐silenced cells undergo phenotypic alteration during sustained culture in terms of losing the well‐differentiated epithelial phenotype of hepatocytes and acquiring dedifferentiated and stem cell‐like characteristics.

### Changes in expression of adhesion molecules

**Figure 3:**
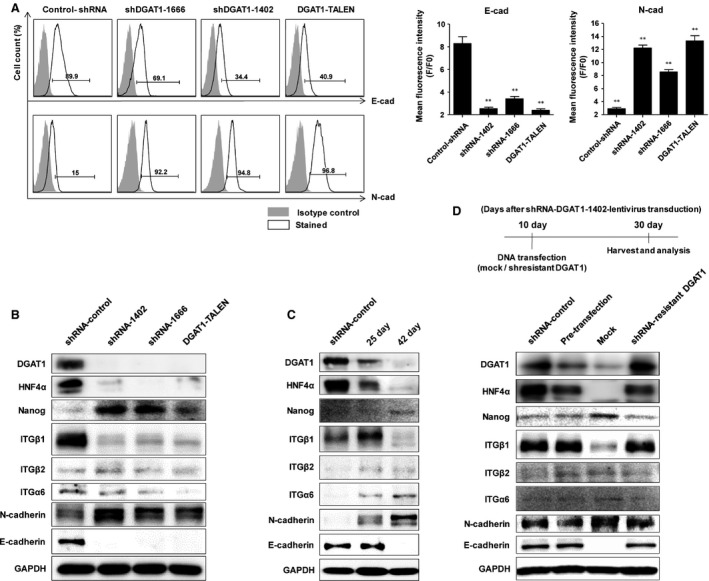
Changes in expression of adhesion molecules in DGAT1‐silenced Huh‐7.5 cell lines. (**A**) Surface expression of N‐ and E‐cadherin in each cell line was examined by flow cytometry. Mean fluorescence intensities (MFIs) of N‐ and E‐cadherin were analysed and are shown as bar graphs. Data are representative of three independent experiments and presented as means ± S.E.M.; asterisks indicate significant differences by anova, **P < 0.01. (**B**) Cells were cultured in complete medium and soluble lysates of each cell line were subjected to immunoblotting for DGAT1, HNF4α, Nanog, integrin β1, integrin β2, integrin α6, E‐cadherin, N‐cadherin, and GAPDH. (**C**) During establishment of a stable cell line, temporal changes in protein levels were examined after transduction of shRNA‐DGAT1‐1666‐lentivirus into Huh‐7.5 cells. The cells were harvested at 25 and 42 days after transduction and soluble lysates were subjected to immunoblotting for DGAT1, HNF4α, Nanog, integrin β1, integrin β2, integrin α6, E‐cadherin, N‐cadherin and GAPDH. (**D**) Ten days after shDGAT1‐1402‐lentivirus transduction in Huh‐7.5 cells, mock vector or shResistant DGAT1 vector was transfected to these DGAT1‐silenced cells. Twenty days after DNA transfection (30 days after DGAT1 silencing), cells were harvested and immunoblotting was done to confirm the expression level of proteins.

Because DGAT1‐silenced cells easily aggregated and formed spheroids, we evaluated the surface expression of cadherins, which play an important role in cell–cell interactions. The expression of E‐cadherin was significantly reduced in DGAT1‐silenced Huh‐7.5 cells, whereas that of N‐cadherin was up‐regulated (Fig. 3A and B). The expression level of integrin β1, an adhesion molecule that contributes to cell‐extracellular matrix (ECM) or cell‐substrate adhesion, was significantly reduced in DGAT1‐silenced cells compared to the control, but the expression level of other integrins was not changed significantly (Fig. 3B). We evaluated the expression of HNF4α as a marker of hepatocyte differentiation. As we have reported previously, DGAT1‐silenced cells showed markedly diminished levels of HNF4α, suggesting a dedifferented state 7. Expression of Nanog, a transcription factor which is involved in self‐renewal of undifferentiated embryonic stem cells and widely used as a marker for stemness, was increased in DGAT1‐silenced cells, supporting that these cells acquired a dedifferentiated and stem‐like phenotype during long‐term silencing of DGAT1 (Fig. 3B and C). Similar to our previous report, adhesion molecule expression was altered at 25 days after shRNA–DGAT1–lentivirus transduction; indeed, the changes were more prominent at 42 days after transduction (Fig. 3C). Diacylglycerol acyltransferase‐1 inhibition with chemical inhibitor (A922500), however, did not cause rapid proliferation (Fig. S2A) or altered expression in adhesion molecules (Fig. S2B). To exclude any off‐target effects caused during silencing of *DGAT1* gene, we transfected shRNA‐resistant *DGAT1* gene to shRNA–DGAT1‐transduced cells 10 days after DGAT1 silencing. By forced expression of *DGAT1* gene, DGAT1 expression was recovered and the expression of HNF4α, Nanog, integrin β1 and cadherins was also maintained (Fig. 3D).

**Figure 4:**
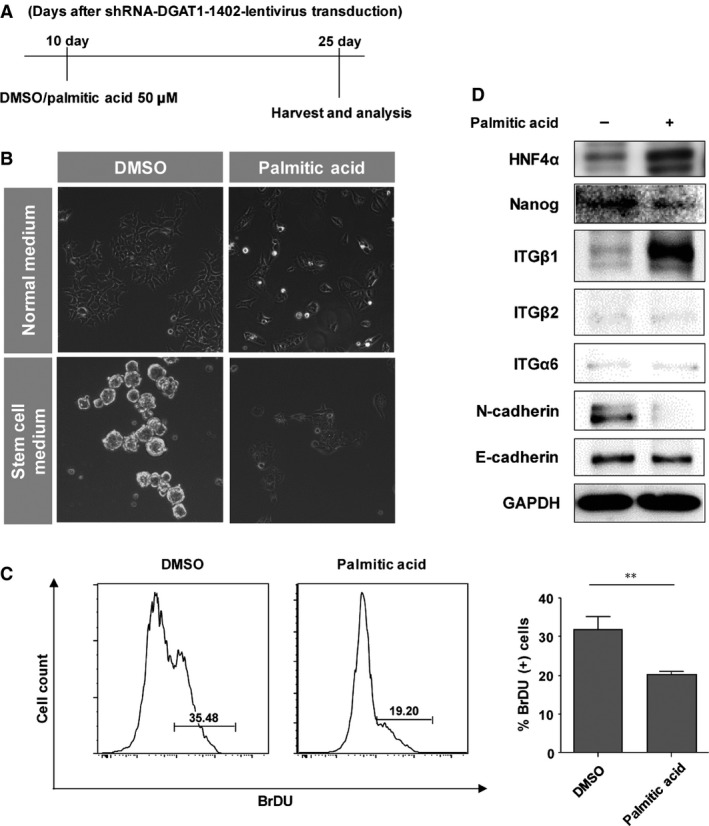
Effect of exogenous palmitic acid treatment after DGAT1 silencing. (**A**) Cells were incubated in medium supplemented with 10% charcoal‐stripped foetal bovine serum and 1% bovine serum albumin with either 50 μM palmitic acid or DMSO from day 10 after transduction with shRNA‐DGAT1‐1666‐lentivirus. (**B**) Cells were cultured in either complete medium or stem cell medium without serum. After 48 hrs incubation, cells were visualized using a phase‐contrast microscope. (**C**) Proliferation of each cell line was measured by BrdU incorporation assay. BrdU‐positive cells were counted by flow cytometry. Histograms are representative of three independent experiments and the proportions (%) of BrdU‐positive cells are shown as with bar graphs. Data are presented as means ± S.E.M.; asterisks indicate significant differences by Student's *t*‐test; **P < 0.01. (**D**) The cells were harvested at 25 days after transduction (days 1 and 15 of palmitic acid treatment) and soluble lysates were subjected to immunoblotting for HNF4α, Nanog, integrin β1, integrin β2, integrin α6, E‐cadherin, N‐cadherin and GAPDH.

We next tested whether these alterations including aggregate formation and high proliferation rate in DGAT1‐silenced Huh‐7.5 cells can be rescued by exogenous administration of palmitic acid. We administered low concentration of BSA‐conjugated palmitic acid (C16:0) to shRNA‐DGAT1‐transduced cells 10 days after the transduction for an additional 15 days (Fig. 4A). Exogenous treatment of palmitic acid treatment significantly suppressed the aggregate formation and proliferation rate of DGAT1‐silenced cells (Fig. 4B and C). Expression levels of HNF4α, Nanog and adhesion molecules were also reversed by palmitic acid treatment (Fig. 4D).

**Figure 5:**
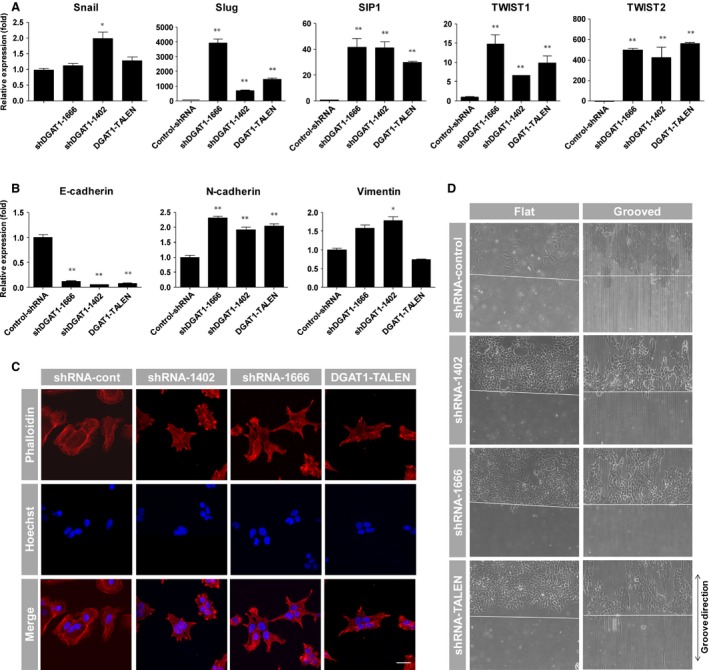
Verification of phenotype related to epithelial–mesenchymal transition. mRNA levels of transcription factors, including Snail, Slug, SIP1, TWIST1, TWIST2 (**A**) and E‐cadherin, N‐cadherin and vimentin (**B**) were examined by real‐time quantitative PCR in each cell line and normalized to β‐actin (n = 3). Data are presented as means ± S.E.M.; asterisks indicate significant differences by anova, *P < 0.05, **P < 0.01. (**C**) DGAT1‐silenced cell lines and the control cell line were fixed and stained for F‐actin (red) with rhodamine‐conjugated phalloidin; nuclei were stained with Hoechst 33342 (blue). Images of actin arrangement and nuclei were obtained by confocal and two‐photon microscopy; scale bar, 30 μm. (**D**) Flat and micropatterned PDMS substrates were prepared with an additional small piece of PDMS to generate a cell‐free area. Cells of each cell line were seeded and cultured on both flat and micropatterned substrates for 24 hrs; small PDMS pieces were then removed to induce cell migration. Images were acquired after an additional 24 hrs.

Because cadherins are also major inducers of the epithelial–mesenchymal transition (EMT), we evaluated whether the phenotypic alteration by DGAT1 silencing was related to the EMT. As expected, mRNA levels of major transcription factors [Snail, Slug, SIP1 (Smad interacting protein 1), TWIST1 and 2 (Twist‐related protein 1 and 2)] and proteins (N‐cadherin, vimentin) associated with EMT 11 were up‐regulated (Fig. 5A and B). The morphology of cells changed to a star‐like shape, and their size was decreased, after DGAT1 silencing. F‐actin distribution in the cytosol was also altered and localized at the periphery of DGAT1‐silenced cell lines (Fig. 5C). These results suggest preferential changes towards a mesenchymal phenotype from the epithelial phenotype; however, cell motility was not increased after DGAT1 silencing, even the cells were cultured on micropatterned substrates (Fig. 5D), which can induce cell migration 12.

To reinforce general significance, we examined spheroid formation, proliferation and expression level of adhesion molecules after DGAT1 silencing in another hepatoma cell line, HepG2 cells. In results, the phenotypic alterations were similarly observed in DGAT1‐silenced HepG2 cells (Fig. S1).

### DGAT1 expression in human liver samples

**Figure 6:**
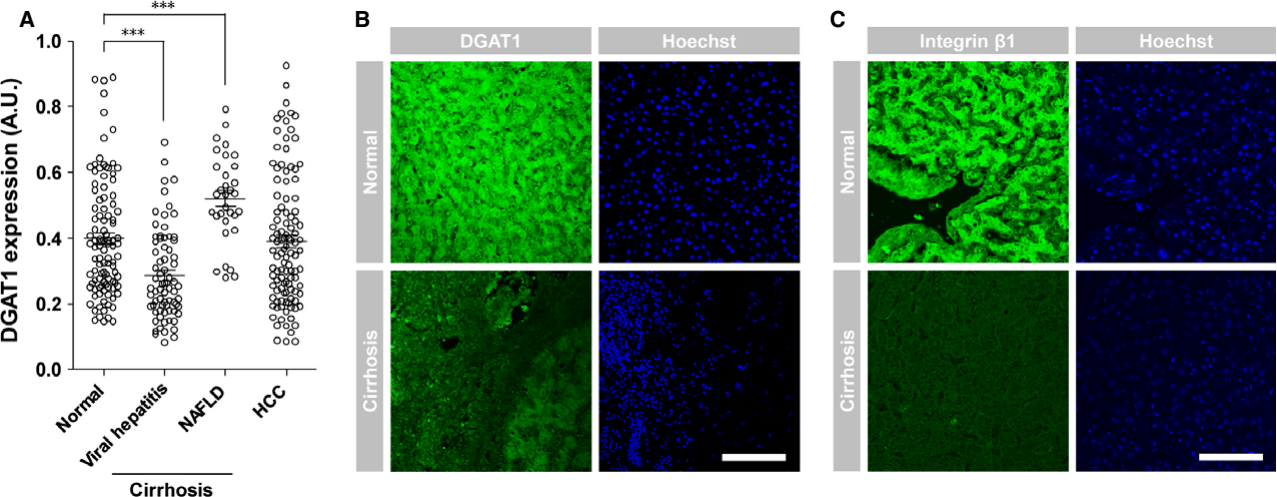
DGAT1 expression in human liver diseases. (**A**) DGAT1 mRNA levels in various liver diseases, including non‐alcoholic fatty liver disease (NAFLD)‐related cirrhosis, viral hepatitis‐related cirrhosis and hepatocellular carcinoma (HCC), were compared to those in the normal liver. DGAT1 expression was down‐regulated in viral hepatitis‐related cirrhosis (*P* < 0.001). Each mark indicates an mRNA level of a patient. Data are presented as means ± S.E.M.; asterisks indicate significant differences by Student's unpaired *t*‐test, ***P < 0.001. Tissue microarray slides containing normal livers and nodular cirrhotic livers without fatty degeneration were stained for DGAT1 (**B**) or integrin β1 (**C**) using Alexa Fluor 488 IgG (green); nuclei were stained with Hoechst 33342 (blue). Tissue IDs of Normal are Dlv06N007 and Dlv06N010 in each; Tissue IDs of Cirrhosis are Dlv062356 and Dlv051170 in each. Images of each sample were obtained by confocal and two‐photon microscopy. Scale bar, 500 μm.

To determine the clinical relevance of DGAT1 silencing, we first analysed several data sets of various human liver diseases in the GEO, a public gene expression profile database. Expression levels were normalized by the UPC method to enable comparison among independent data sets 13. Diacylglycerol acyltransferase‐1 expression was markedly down‐regulated in viral hepatitis‐related cirrhotic livers compared to normal livers, whereas its expression was elevated in non‐alcoholic fatty liver disease‐related cirrhosis (Fig. 6A) as reported previously 14, 15. Tissue microarray staining revealed reduced expression of DGAT1 (Fig. 6B) and integrin β1 (Fig. 6C) in the nodular cirrhotic liver without fatty degeneration compared to normal liver. Collectively, these results indicate that down‐regulation of DGAT1 and integrin β1 may be involved in the progression of liver cirrhosis without fatty degeneration.

## Discussion

In this study, we discovered that complete and long‐term silencing of DGAT1 directed two different hepatoma cell lines, Huh‐7.5 and HepG2, towards a dedifferentiated and stem cell‐like phenotype. Diacylglycerol acyltransferase‐1‐silenced hepatoma cell lines lost their hepatocyte‐specific functions, including albumin secretion, and well‐differentiated epithelial characteristics. Diacylglycerol acyltransferase‐1‐silenced cell lines tended to form aggregates in the presence of high levels of growth factors, similar to other stem cells, possibly because of the changes in the expression of adhesion molecules. The phenotypic alteration induced by DGAT1 depletion was not observed by the treatment of DGAT1 inhibitor. These discrepant results by two different methods, gene silencing or chemical inhibition, might be caused by the establishment of complete and long‐term silencing of DGAT1 by gene silencing method.

Currently, stem cell‐like features in non‐stem cells, such as cancer cells, have included self‐renewal, tumourigenesis, angiogenesis, multilineage differentiation and chemo‐/radio‐resistance 16, 17, 18. In this study, we identified stem cell‐like characteristics in DGAT1‐silenced Huh‐7.5 cell lines. The stem cell‐like behaviours caused by complete and long‐term silencing of DGAT1 were also observed in another hepatocellular carcinoma cell line, HepG2. This stem cell‐like phenotype in DGAT1‐silenced cell lines may be related to the dedifferentiation of hepatocytes. HNF4α 19, 20, 21 and integrin β1 22, 23 are known to play critical roles in the regulation of the hepatic differentiation state. We have confirmed that HNF4α and integrin β1 expression was down‐regulated in DGAT1‐silenced cell lines.

The phenotypic alteration may also be related to the EMT. Generally, the EMT is accompanied by the dissociation of cell–cell junctions, cytoskeletal rearrangements, increased cell motility and synthesis of ECM. Alteration of transcription factors eventually results in the acquisition of a mesenchymal phenotype 24. We modulated the phenotype of vascular smooth muscle cells by culturing on a micropatterned substrate in a previous study 12. In addition to the alteration of protein expression, cell motility along the parallel pattern on the microgrooved substrate was also increased 12. Although the motility of DGAT1‐silenced cell lines was not elevated by the micropatterned substrate, morphological changes and cytoskeletal rearrangement were observed. Expressions of transcription factors, such as snail, slug, SIP1 and TWIST 1, 2; and junctional proteins, such as E‐cadherin, N‐cadherin and vimentin, were altered towards the EMT process. The EMT has been reported to generate stem cell traits in human and murine mammalian epithelial cells 25 and cancer cells 26, 27, 28, 29. Therefore, DGAT1 silencing could induce changes in the expression of differentiation and EMT markers, which may lead to the acquisition of a stem cell‐like phenotype.

Diacylglycerol acyltransferase‐1 is known to be involved in the trafficking of HCV core proteins to the lipid droplet 6. In a previous study, we showed that DGAT1 silencing also impaired HCV entry by claudin‐1 down‐regulation 7. Fatty acid homoeostasis alteration occurred in DGAT1‐silenced cell lines; expressions of claudin‐1 and HNF4α were affected by the altered intracellular lipid homoeostasis; indeed, restoration of intracellular fatty acid homoeostasis by exogenous palmitic acid resulted in recovery of claudin‐1 and HNF4α expression. Likewise, in this study, we demonstrated that low‐dose, exogenous palmitic acid treatment rescued the cells from the phenotypic change. Therefore, altered fatty acid homoeostasis may be responsible for dedifferentiation after DGAT1 silencing. Indeed, a previous study revealed that DGAT1 recycles the products of TG hydrolysis, which are partial glycerides, for TG synthesis, meaning that DGAT1 is involved not only in TG biosynthesis but also in the overall regulation of intracellular lipid status at least in human hepatoma cells 30. Thus, DGAT1 inhibition may decrease the intracellular lipid pool, which comprises TGs, partial glycerides and free fatty acids, in human hepatoma cells 30, 31. A remaining question is the mechanism by which disruption of intracellular lipid homoeostasis leads to hepatic dedifferentiation.

Diacylglycerol acyltransferase‐1 is known to be involved in hepatic steatosis 32 and is considered a therapeutic target for various metabolic diseases, such as obesity and type 2 diabetes mellitus 33, 34. Diacylglycerol acyltransferase‐1 deficiency in mice was reported to cause resistance to obesity and hepatic steatosis 35, 36. However, in a recent study, a loss‐of‐function mutation in DGAT1 was associated with fatal congenital diarrhoea in humans, raising concern over the use of DGAT1 inhibitors 5. In this study, we demonstrated that DGAT1 silencing of human hepatocytes might lead to dysregulated fatty acid metabolism and alteration of cellular differentiation status.

Diacylglycerol acyltransferase enzymes involve DGAT1 and DGAT2. Even though DGAT2 is also involved in the final step of TG biosynthesis, its silencing did not induce dedifferentiated phenotype in both Huh‐7.5 and HepG2 cells. Diacylglycerol acyltransferase‐1 and DGAT2 have been reported that they function non‐redundantly in human hepatocytes 33: DGAT1 acts in the re‐esterification of partial glycerides formed by intracellular lipolysis, using preformed fatty acids; whereas DGAT2 participates in *de novo* synthesis of TG acting at upstream of DGAT1. Diacylglycerol acyltransferase‐1 is expressed in both liver and small intestine in human, additionally murine DGAT1 is barely expressed in liver, but expressed mostly in small intestine 5. Collectively, it can be inferred that DGAT1 and DGAT2 have different roles, and DGAT1 takes critical role of maintaining intracellular lipid homoeostasis in human hepatoma cells.

Our findings of phenotypic alteration by DGAT1 silencing were identified in Huh‐7.5 and HepG2 cell lines. Because these cell lines are originated from liver cancers, our results may not precisely reflect the actual effects of DGAT1 silencing in normal hepatocytes. Hence, this effect should be confirmed in primary human hepatocytes. Again, the underlying mechanisms how human hepatoma cells become dedifferentiated after DGAT1‐silencing remains to be elucidated.

## Conflicts of interest

The authors confirm that there are no conflicts of interest.

## Supporting information


**Figure S1** Phenotypic alterations induced by DGAT1 silencing in HepG2 cells.

**Figure S2** No significant effect of DGAT1 inhibitor on cellular phenotype change in Huh‐7.5 cells.

## References

[1] Cases S , Smith SJ , Zheng Y‐W , *et al* Identification of a gene encoding an acyl CoA: diacylglycerol acyltransferase, a key enzyme in triacylglycerol synthesis. Proc Natl Acad Sci USA. 1998; 95: 13018–23. 9789033 10.1073/pnas.95.22.13018 PMC23692

[2] Yen C‐LE , Stone SJ , Koliwad S , *et al* Thematic review series: glycerolipids. DGAT enzymes and triacylglycerol biosynthesis. J Lipid Res. 2008; 49: 2283–301. 18757836 10.1194/jlr.R800018-JLR200 PMC3837458

[3] Smith SJ , Cases S , Jensen DR , *et al* Obesity resistance and multiple mechanisms of triglyceride synthesis in mice lacking Dgat. Nat Genet. 2000; 25: 87–90. 10802663 10.1038/75651

[4] DeVita RJ , Pinto S . Current status of the research and development of diacylglycerol O‐acyltransferase 1 (DGAT1) inhibitors: miniperspective. J Med Chem. 2013; 56: 9820–5. 23919406 10.1021/jm4007033

[5] Haas JT , Winter HS , Lim E , *et al* DGAT1 mutation is linked to a congenital diarrheal disorder. J Clin Invest. 2012; 122: 4680. 23114594 10.1172/JCI64873 PMC3533555

[6] Herker E , Harris C , Hernandez C , *et al* Efficient hepatitis C virus particle formation requires diacylglycerol acyltransferase‐1. Nat Med. 2010; 16: 1295–8. 20935628 10.1038/nm.2238 PMC3431199

[7] Sung PS , Murayama A , Kang W , *et al* Hepatitis C virus entry is impaired by claudin‐1 downregulation in diacylglycerol acyltransferase‐1 (DGAT1)‐deficient cells. J Virol. 2014; 88: 9233–44. 24899196 10.1128/JVI.01428-14 PMC4136266

[8] Delhaye M , Louis H , Degraef C , *et al* Relationship between hepatocyte proliferative activity and liver functional reserve in human cirrhosis. Hepatology. 1996; 23: 1003–11. 8621125 10.1053/jhep.1996.v23.pm0008621125

[9] Wang Z , Liu F , Tu W , *et al* Embryonic liver fodrin involved in hepatic stellate cell activation and formation of regenerative nodule in liver cirrhosis. J Cell Mol Med. 2012; 16: 118–28. 21388516 10.1111/j.1582-4934.2011.01290.x PMC3823098

[10] Ramirez‐Zacarias JL , Castro‐Munozledo F , Kuri‐Harcuch W . Quantitation of adipose conversion and triglycerides by staining intracytoplasmic lipids with Oil red O. Histochemistry. 1992; 97: 493–7. 1385366 10.1007/BF00316069

[11] Kang Y , Massagué J . Epithelial‐mesenchymal transitions: twist in development and metastasis. Cell. 2004; 118: 277–9. 15294153 10.1016/j.cell.2004.07.011

[12] Chang S , Song S , Lee J , *et al* Phenotypic modulation of primary vascular smooth muscle cells by short‐term culture on micropatterned substrate. PLoS ONE. 2014; 9: e88089. 24505388 10.1371/journal.pone.0088089 PMC3913720

[13] Piccolo SR , Withers MR , Francis OE , *et al* Multiplatform single‐sample estimates of transcriptional activation. Proc Natl Acad Sci USA. 2013; 110: 17778–83. 24128763 10.1073/pnas.1305823110 PMC3816418

[14] Nakamuta M , Kohjima M , Morizono S , *et al* Evaluation of fatty acid metabolism‐related gene expression in nonalcoholic fatty liver disease. Int J Mol Med. 2005; 16: 631–5. 16142397

[15] Kohjima M , Enjoji M , Higuchi N , *et al* Re‐evaluation of fatty acid metabolism‐related gene expression in nonalcoholic fatty liver disease. Int J Mol Med. 2007; 20: 351–8. 17671740

[16] Reya T , Morrison SJ , Clarke MF , *et al* Stem cells, cancer, and cancer stem cells. Nature. 2001; 414: 105–11. 11689955 10.1038/35102167

[17] Pardal R , Clarke MF , Morrison SJ . Applying the principles of stem‐cell biology to cancer. Nat Rev Cancer. 2003; 3: 895–902. 14737120 10.1038/nrc1232

[18] Clevers H . The cancer stem cell: premises, promises and challenges. Nat Med. 2011; 17: 313–9. 21386835 10.1038/nm.2304

[19] Watt AJ , Garrison WD , Duncan SA . HNF4: a central regulator of hepatocyte differentiation and function. Hepatology. 2003; 37: 1249–53. 12774000 10.1053/jhep.2003.50273

[20] Kamiya A , Inoue Y , Gonzalez FJ . Role of the hepatocyte nuclear factor 4α in control of the pregnane X receptor during fetal liver development. Hepatology. 2003; 37: 1375–84. 12774017 10.1053/jhep.2003.50212

[21] Parviz F , Matullo C , Garrison WD , *et al* Hepatocyte nuclear factor 4α controls the development of a hepatic epithelium and liver morphogenesis. Nat Genet. 2003; 34: 292–6. 12808453 10.1038/ng1175

[22] Couvelard A , Bringuier A‐F , Dauge M‐C , *et al* Expression of integrins during liver organogenesis in humans. Hepatology. 1998; 27: 839–47. 9500715 10.1002/hep.510270328

[23] Weinstein M , Monga SPS , Liu Y , *et al* Smad proteins and hepatocyte growth factor control parallel regulatory pathways that converge on β1‐integrin to promote normal liver development. Mol Cell Biol. 2001; 21: 5122–31. 11438667 10.1128/MCB.21.15.5122-5131.2001 PMC87237

[24] Radisky DC . Epithelial‐mesenchymal transition. J Cell Sci. 2005; 118: 4325–6. 16179603 10.1242/jcs.02552

[25] Mani SA , Guo W , Liao M‐J , *et al* The epithelial‐mesenchymal transition generates cells with properties of stem cells. Cell. 2008; 133: 704–15. 18485877 10.1016/j.cell.2008.03.027 PMC2728032

[26] Zhu L‐F , Hu Y , Yang C‐C , *et al* Snail overexpression induces an epithelial to mesenchymal transition and cancer stem cell‐like properties in SCC9 cells. Lab Invest. 2012; 92: 744–52. 22349639 10.1038/labinvest.2012.8

[27] Kurrey NK , Jalgaonkar SP , Joglekar AV , *et al* Snail and slug mediate radioresistance and chemoresistance by antagonizing p53‐mediated apoptosis and acquiring a stem‐like phenotype in ovarian cancer cells. Stem Cells. 2009; 27: 2059–68. 19544473 10.1002/stem.154

[28] Fuxe J , Vincent T , de Herreros AG . Transcriptional crosstalk between TGFβ and stem cell pathways in tumor cell invasion: role of EMT promoting Smad complexes. Cell Cycle. 2010; 9: 2363–74. 20519943 10.4161/cc.9.12.12050

[29] Polyak K , Weinberg RA . Transitions between epithelial and mesenchymal states: acquisition of malignant and stem cell traits. Nat Rev Cancer. 2009; 9: 265–73. 19262571 10.1038/nrc2620

[30] Wurie HR , Buckett L , Zammit VA . Diacylglycerol acyltransferase 2 acts upstream of diacylglycerol acyltransferase 1 and utilizes nascent diglycerides and *de novo* synthesized fatty acids in HepG2 cells. FEBS J. 2012; 279: 3033–47. 22748069 10.1111/j.1742-4658.2012.08684.x

[31] Wurie HR , Buckett L , Zammit VA . Evidence that diacylglycerol acyltransferase 1 (DGAT1) has dual membrane topology in the endoplasmic reticulum of HepG2 cells. J Biol Chem. 2011; 286: 36238–47. 21846726 10.1074/jbc.M111.251900 PMC3196132

[32] Jin J , Iakova P , Breaux M , *et al* Increased expression of enzymes of triglyceride synthesis is essential for the development of hepatic steatosis. Cell Rep. 2013; 3: 831–43. 23499441 10.1016/j.celrep.2013.02.009 PMC3615099

[33] Birch AM , Buckett LK , Turnbull AV . DGAT1 inhibitors as anti‐obesity and anti‐diabetic agents. Curr Opin Drug Discov Dev. 2010; 13: 489–96. 20597032

[34] Cao J , Zhou Y , Peng H , *et al* Targeting Acyl‐CoA: diacylglycerol acyltransferase 1 (DGAT1) with small molecule inhibitors for the treatment of metabolic diseases. J Biol Chem. 2011; 286: 41838–51. 21990351 10.1074/jbc.M111.245456 PMC3308890

[35] Chen HC , Farese RV Jr . Inhibition of triglyceride synthesis as a treatment strategy for obesity: lessons from DGAT1‐deficient mice. Arterioscler Thromb Vasc Biol. 2005; 25: 482–6. 15569818 10.1161/01.ATV.0000151874.81059.ad

[36] Villanueva CJ , Monetti M , Shih M , *et al* Specific role for acyl CoA: diacylglycerol acyltransferase 1 (Dgat1) in hepatic steatosis due to exogenous fatty acids. Hepatology. 2009; 50: 434–42. 19472314 10.1002/hep.22980 PMC3097135

