# Supplementary figures and images for: Prolonged silencing of diacylglycerol acyltransferase‐1 induces a dedifferentiated phenotype in human liver cells
Source: J Cell Mol Med. 2015 Oct 23;20(1):38–47. doi: 10.1111/jcmm.12685 (PMC4717863; doi:10.1111/jcmm.12685)

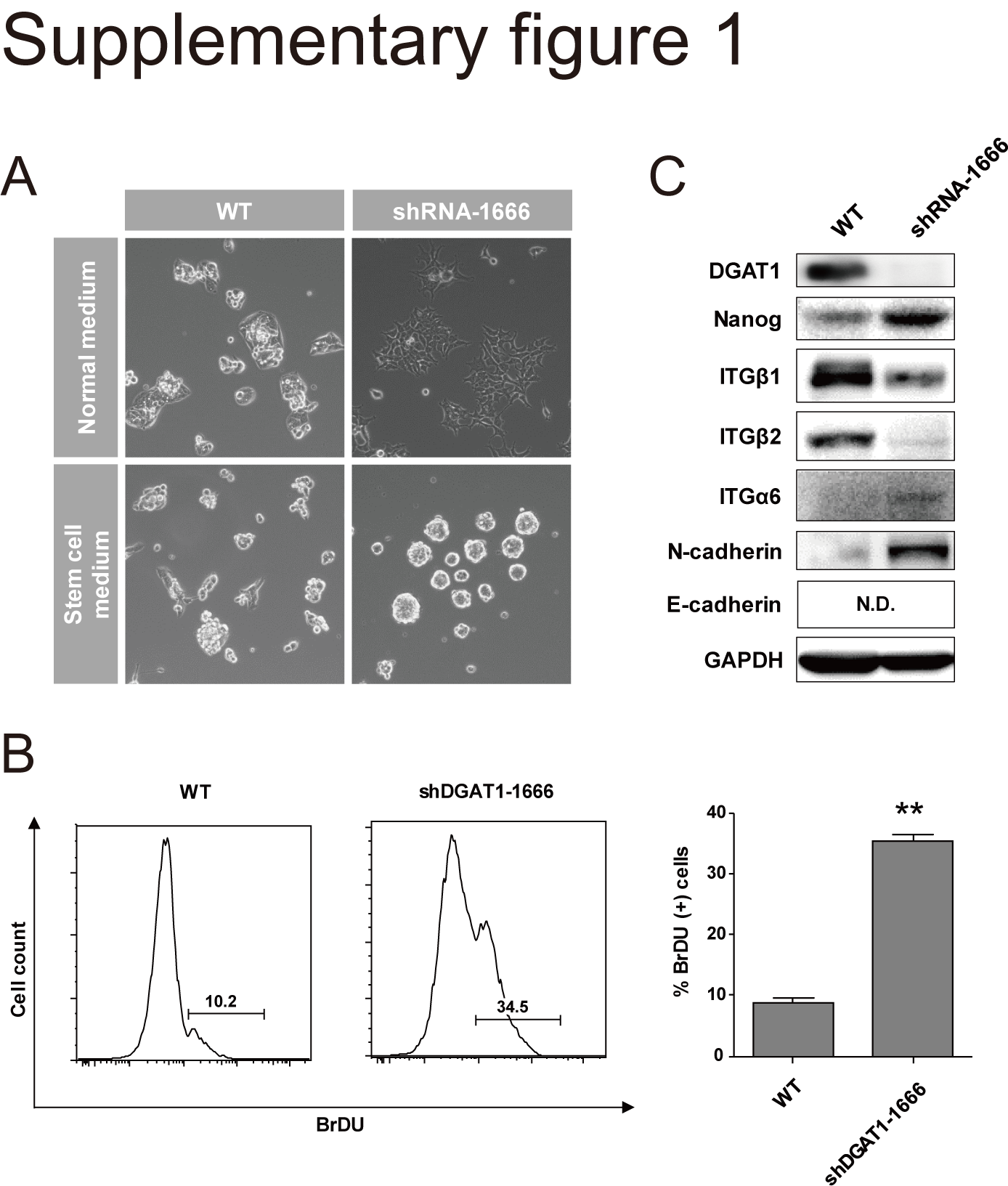

Supplement: Supplementary file 1 — Figure S1 Phenotypic alterations induced by DGAT1 silencing in HepG2 cells. [file JCMM-20-038-s001.tif]

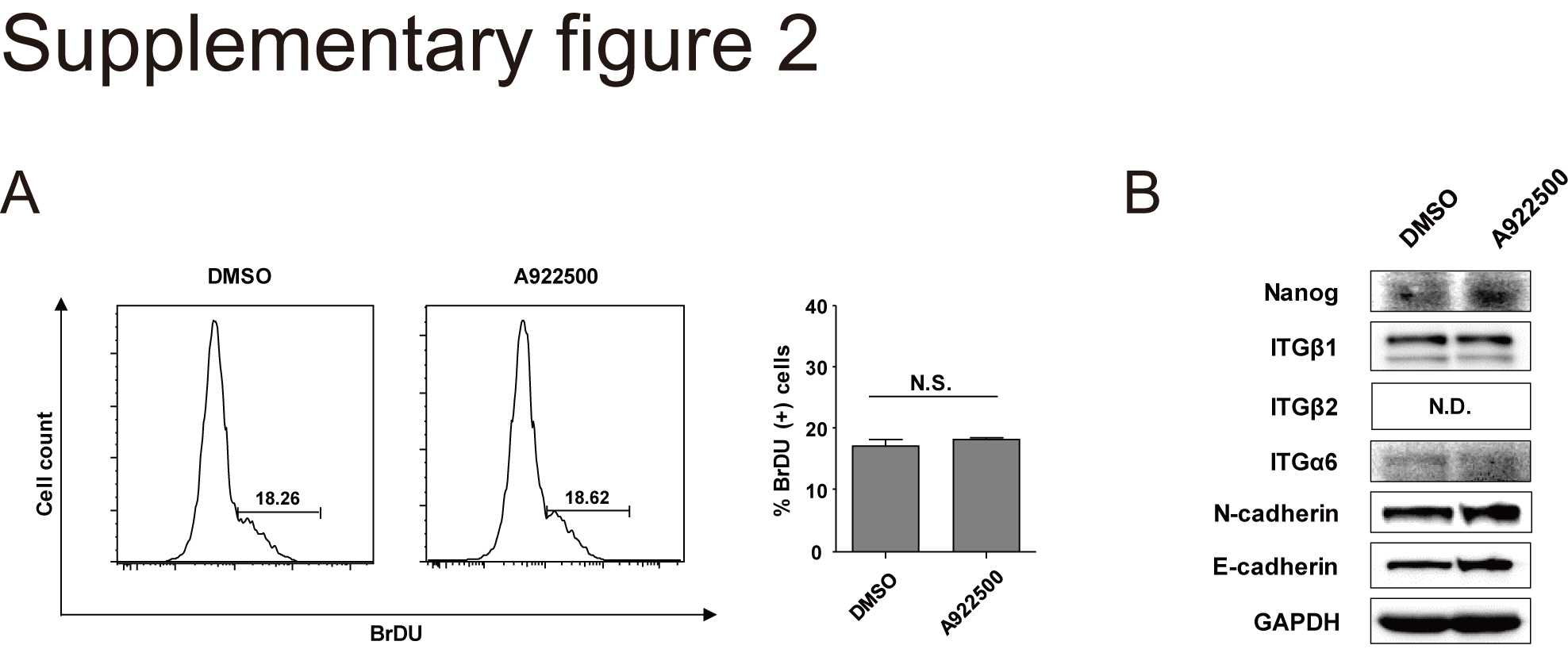

Supplement: Supplementary file 2 — Figure S2 No significant effect of DGAT1 inhibitor on cellular phenotype change in Huh‐7.5 cells. [file JCMM-20-038-s002.tif]
